# Neurosyphilis Presenting as Sleep Disorder and Muscle Soreness Complicated With HIV Infection: A Case Report

**DOI:** 10.1155/crdi/4758423

**Published:** 2025-08-18

**Authors:** Shu-Qi Yang, Hua-Tang Zhang, Yi-Jie Lin, Xing Wang, Yan-Yan Qiu, Xue-Ping Yu

**Affiliations:** ^1^Department of Infection Disease, Clinical Medical Research Center for Bacterial and Fungal Infectious Diseases of Fujian Province, Fujian Medical University Affiliated First Quanzhou Hospital, Quanzhou, Fujian, China; ^2^Department of Key Laboratory of Screening and Control of Infectious Diseases, Quanzhou Medical College, Quanzhou, Fujian, China

**Keywords:** HIV, muscle soreness, neurosyphilis, sexually transmitted disease, sleep disorder

## Abstract

Neurosyphilis is a tertiary syphilis complication or manifestation caused by *Treponema pallidum,* which invades the central nervous system. Its clinical manifestations are often nonspecific. Neurosyphilis symptoms frequently resemble those of spinal cord diseases and mental disorders, posing significant challenges for early diagnosis. With improving living standards and unprotected sexual behaviors becoming more common, the global incidence of syphilis has increased, along with a concurrent rise in reports of neurosyphilis, particularly among individuals with HIV infection. Although some patients with neurosyphilis mainly present with brain-related symptoms, it is rare for sleep disorders and general muscle soreness to be the predominant manifestations. This paper presents a case of neurosyphilis complicated by HIV coinfection, manifesting as sleep disorder and muscle soreness, providing insight into the impact of syphilis infection on the nervous system.

## 1. Presentation

A 65-year-old man with a history of unprotected sexual encounters but no prior sexually transmitted infections (STIs) was admitted to our hospital, complaining of muscle aches and sleep disturbances for more than 10 days. He experienced systemic muscle soreness for over 10 days without any obvious cause, and resting did not provide relief. He also reported fatigue and sleep disorders, characterized by difficulty falling asleep, involuntary imaginations, and reduced sleep quality, often accompanied by vivid dreams. Six days prior to admission, he went to Community Hospital, where an HIV antibody screening returned positive, leading to his transfer to our hospital. He had a history of diabetes mellitus for over a decade and was treated with oral medications including metformin, glimepiride, and canagliflozin. Random blood glucose measurements during admission fluctuated between 6 and 9 mmol/L (normal range: 3.9–11.1 mmol/L), indicating stable glycemic control without acute hyperglycemia.

## 2. Investigations

On physical examination, the patient was conscious but appeared listless. There was no jaundice of the skin or mucous membranes. Cardiopulmonary examination was unremarkable, and abdominal examination revealed no tenderness or rebound pain. Muscle examination showed no localized tenderness, erythema, or swelling, and systemic muscle soreness was documented as a subjective symptom without objective signs suitable for imaging. Routine blood, urine, and stool tests were unremarkable; liver and kidney function tests were within normal limits; and head CT showed no abnormal imaging findings. Routine blood tests revealed random blood glucose levels of 6–9 mmol/L, consistent with stable glycemic control. Glycated hemoglobin (HbA1c) was not performed due to the acute nature of the admission (neurosyphilis with HIV coinfection), though the absence of diabetic complications (e.g., ketosis) supported the clinical assessment of glucose management. Serological findings support the diagnosis of syphilis. Nontreponemal tests—the toluidine red unheated serum test (TRUST) and the rapid plasma reagin (RPR) assay—are weakly positive at low titre (TRUST reactive, RPR 1:1). The treponemal-specific *Treponema pallidum* particle agglutination assay (TPPA) is positive, confirming infection with *Treponema pallidum*. HIV diagnostic tests were also positive, with a CD4 cell count of 262.55 cells/μL (normal range: 500–1500 cells/μL). This CD4 count, combined with the absence of AIDS-defining illnesses (e.g., *Pneumocystis* pneumonia or Kaposi sarcoma), indicated the patient was in the asymptomatic stage (clinical stage II) of HIV infection. After admission, he received symptomatic treatment, including acid suppression, analgesia, and estazolam as a sleep aid, but his symptoms did not improve significantly. Specific medications included nizatidine and aluminum-magnesium mixture for gastrointestinal protection; celecoxib and eperisone for analgesia; and prednisone 10 mg and mecobalamin for neuroinflammation control and nerve nutrition. Diabetes management continued with metformin, glimepiride, and canagliflozin, supplemented by atorvastatin, clopidogrel, lactulose, and vitamin C for comorbidity support. Despite these interventions, his symptoms persisted, prompting cerebrospinal fluid (CSF) analysis. CSF analysis showed a total nucleated cell count of 65 × 10^6^/L (normal range: 0–5 × 10^6^/L), protein quantification of 2131 mg/L (normal range: 150–450 mg/L), and a weakly positive TPPA, confirming neurosyphilis. Cytological examination revealed 98.5% mononuclear cells and 1.5% polymorphonuclear cells, consistent with chronic inflammatory changes in the central nervous system (see Supporting Table 1).

## 3. Treatment

The patient was treated with 4 million units of penicillin G sodium every 6 h. The combination of specific antisyphilitic therapy and adjunctive medications led to rapid symptom improvement, with reduced muscle soreness and improved sleep quality noted within 48 h. By the fourth day, the muscle soreness had resolved, and he was able to sleep soundly without vivid dreams. After 15 days of continuous treatment, 240 units of benzathine penicillin were administered intramuscularly. On the 22nd day, a repeat CSF examination revealed a negative TPPA titer (Table 1), and his symptoms continued to improve post-discharge. Notably, antiretroviral therapy (ART) was not initiated during the patient's hospitalization. The primary focus was on completing the neurosyphilis treatment regimen, which consisted of penicillin G sodium (4 million units every 6 h for 15 days), followed by a single intramuscular injection of benzathine penicillin. A comprehensive evaluation for ART, including assessments of liver and kidney function, as well as patient counseling on treatment adherence, was planned to be conducted after discharge. Following discharge, the patient's HIV RNA load was 1.32 × 10^4^ copies/mL, indicating the necessity for opportunistic infection prophylaxis and ART. The ART regimen, prescribed by HIV specialists according to current guidelines, consisted of tenofovir disoproxil fumarate + emtricitabine + dolutegravir, with scheduled follow-up every 3 months for viral load and CD4 count monitoring.

## 4. Discussion

Neurosyphilis-induced sleep disorders are associated with brain dysfunction, particularly in regions responsible for regulating the sleep–wake cycle [1]. In cases of neurosyphilis co-occurring with HIV infection, some patients experience complete system resolution post-treatment [2]. In this case, the patient showed significant symptom improvement following standard therapy, underscoring the critical nature of prompt diagnosis and appropriate treatment. Notably, the patient's HIV infection was in the asymptomatic stage (CD4 count: 262.55 cells/μL), which allowed prioritization of neurosyphilis treatment over immediate ART initiation. This approach aligns with guidelines recommending sequential management of coinfections to avoid therapeutic conflicts [3]. It should be noted that CSF sample testing was delayed due to transportation and preprocessing, potentially affecting the timeliness of cell count results. Although the laboratory data were double-validated and consistent with clinical findings, future studies will adhere to the 1-hour post-collection testing protocol to minimize fluctuations. Penicillin is the first choice for the treatment of neurosyphilis, but it has been reported that combining ceftriaxone sodium with penicillin may be more effective than penicillin alone [4]. For patients with neurosyphilis combined with HIV infection, the threshold for abnormal CSF WBC count should be adjusted to > 20 × 10^6^/L to enhance the diagnostic accuracy [5]. Some researchers have proposed that a CSF WBC count of > 10 × 10^6^/L after regular ART should be considered a diagnostic criterion for neurosyphilis in the context of HIV coinfection [6].

The nonspecific clinical presentation of patients with concurrent HIV and neurosyphilis complicates early diagnosis and treatment. Diagnosis typically relies on patient history, clinical signs, and CSF analysis [3]. It should be noted that the patient had a 10-year history of diabetes mellitus, and its complications such as peripheral neuropathy or myopathy might cause muscle soreness and sleep disorders. However, the patient had well-controlled blood glucose, the generalized muscle soreness lacked distal symmetric characteristics, and both muscle enzyme tests (normal creatine kinase) and head CT were unremarkable, ruling out a diabetic etiology. Additionally, HIV infection could induce neuroinflammation through immune activation to cause similar symptoms, but the patient's CD4 count was 262.55 cells/μL (not severely suppressed), ART had not been initiated, and there was no cognitive impairment, which did not support HIV-related neuropathy. Meanwhile, the diabetic medications (metformin, glimepiride, etc.) rarely caused myalgia side effects, and symptomatic treatment with estazolam showed no significant improvement, excluding drug factors and pure psychogenic causes. The diagnosis of neurosyphilis was confirmed by CSF examination revealing positive TPPA, increased protein quantification, and leukocyte count, combined with the remarkable symptom improvement within 48 h after penicillin treatment. This case highlights that for patients with comorbid HIV and diabetes, comprehensive differential diagnosis should be based on clinical manifestations, laboratory tests, and treatment response, with particular emphasis on CSF etiological analysis to avoid delayed diagnosis due to nonspecific symptoms. In this case, the main symptoms were systemic muscle soreness and sleep disorders, which are rarely reported. However, the suboptimal response to drug therapy, along with a positive serological test for syphilis, coupled with the CSF findings of increased protein and WBC counts, substantiated the diagnosis of neurosyphilis.

## 5. Learning Point

For patients with previous HIV infection combined with syphilis infection who suddenly develop systemic myalgia and sleep disorders, clinicians should be aware of the possibility of neurosyphilis, and timely syphilis serology and CSF examination are crucial to prevent treatment delays.

## Figures and Tables

**Table 1:** Cerebrospinal fluid routine biochemical parameters in neurosyphilis with HIV coinfection.

Item	Initial presentation	Final presentation
Pandy test	(±)	(+)
Total nucleated cells (× 10^6^/L)	65	40
GLU (mmol/L)	4.7	7.3
Chlorine (mmol/L)	126.3	127.1
Protein quantification (mg/L)	2131	1834
TPPA	(±)	(−)
TRUST	(−)	(−)

## Data Availability

The data used to support the findings of this study are available from the corresponding author upon request.

## Supporting Information

Supporting Table 1 shows the cerebrospinal fluid cytological profiles (including Pandy test, total cellular score, white blood cell count, and cellular differential) in the patient with neurosyphilis and HIV coinfection, comparing initial and final presentations.
