# Supplementary material for: Neurosyphilis Presenting as Sleep Disorder and Muscle Soreness Complicated With HIV Infection: A Case Report
Source: Case Rep Infect Dis. 2025 Aug 18;2025:4758423. doi: 10.1155/crdi/4758423 (PMC12377965; doi:10.1155/crdi/4758423)
Supplement: Supporting Information — Additional supporting information can be found online in the Supporting Information section. [file 4758423.f1.docx]

**Supplementary Table 1: Cerebrospinal Fluid Cytological Profiles in Neurosyphilis with HIV Coinfection**

| Item | Initial presentation | Final presentation |
| --- | --- | --- |
| Pandy Test | （+-） | 1+ |
| Total cellular score (×10⁶/L) | 5026 | 260 |
| White blood cell count (×10⁶/L) | 26 | 260 |
| Cellular Differential | Mononuclear cells: 98.5%,  Polymuclear cells: 1.5% | Mononuclear cells: 95%,  Polymuclear cells: 5% |

Note: Total nucleated cell count includes red and white blood cells. Sample hemolysis may have affected initial counts.
